# Successful underwater endoscopic submucosal dissection with gel immersion for early gastric cancer in an upside-down stomach

**DOI:** 10.1055/a-2275-0894

**Published:** 2024-03-14

**Authors:** Takahiro Muramatsu, Tomoaki Tashima, Tsubasa Ishikawa, Tomonori Kawasaki, Yumi Mashimo, Takao Itoi, Shomei Ryozawa

**Affiliations:** 1 183786 Gastroenterology, Saitama Medical University International Medical Center, Hidaka, Japan; 2 183786 Pathology, Saitama Medical University International Medical Center, Hidaka, Japan; 3 38548 Gastroenterology and Hepatology, Tokyo Medical University Hospital, Shinjuku-ku, Japan

An upside-down stomach (UDS) is a relatively rare type of esophageal hiatal hernia; almost the entire stomach prolapses into the posterior mediastinum in affected patients 1 .

Surgical procedures for gastric cancer arising from a UDS have been reported 2 ; however, no reports on endoscopic submucosal dissection (ESD) for early gastric cancer arising in a UDS are available. In patients with a UDS, endoscope maneuverability is poor because their stomachs are inverted. Recently, water or gel immersion has been reported to be useful for improving the field of view and scope maneuverability 3 4 5 . Herein, we describe underwater ESD with gel immersion performed successfully for early gastric cancer arising from a UDS ( Video 1 ).

Successful underwater endoscopic submucosal dissection with gel immersion for early gastric cancer arising in an upside-down stomach.Video 1

An 85-year-old woman with a UDS ( Fig. 1 **a,b** ) presented with early gastric cancer (lesion size 10 mm, type 0-IIc) located on the lesser curvature of the upper body ( Fig. 2 **a,b** ). Approaching the lesion was difficult due to the UDS; thus, gas was removed from the lumen and replaced with water and gel (Viscoclear; Otsuka Pharmaceutical, Tokushima, Japan) to establish an underwater condition. The water–gel mixture maintained a lower intraluminal pressure and allowed us to approach the lesion, even from its distal side, under a clearer view ( Fig. 3 **a–h** ).

**Fig. 1:**
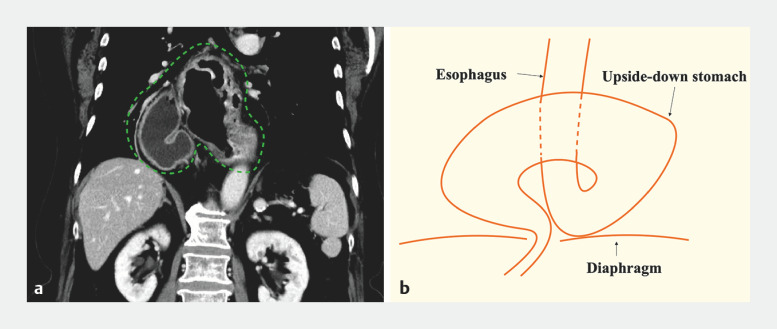
Computed tomography (CT) image and schema of the upside-down stomach (UDS). **a** CT image shows that the entire stomach has prolapsed into the thoracic cavity through the hiatal hernia **b** Schema of the UDS.

**Fig. 2:**
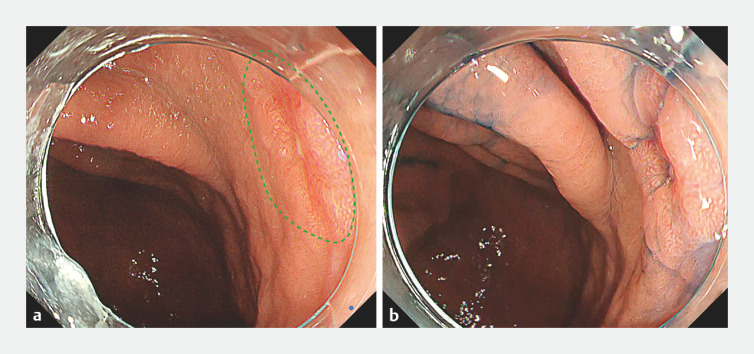
Endoscopic image **a** White light image. Upper gastrointestinal endoscopy shows a depressed lesion (0-IIc) located on the lesser curvature of the upper body (diameter 10 mm). **b** Indigo carmine dye image.

**Fig. 3:**
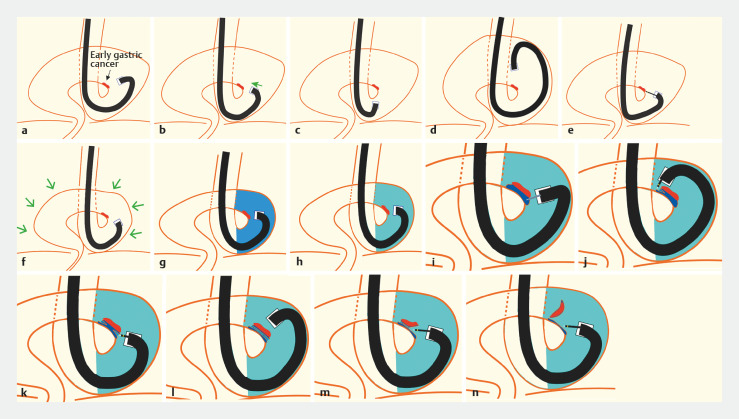
Schema of underwater endoscopic submucosal dissection (ESD) with gel immersion in an upside-down stomach (UDS). **a** View under gas. **b** Attempts to approach the lesion. **c** The endoscope is pulled back. **d** Pushing the endoscope fails to achieve access to the lesion. **e** Markings around the lesion. **f** Removal of the gas from the lumen. **g** Underwater view. **h** View under combined water and gel (Viscoclear; Otsuka Pharmaceutical, Tokushima, Japan) immersion; the conditions established allow access to the lesion. **i** Local injection. **j** Initial mucosal incision is made on the distal edge of the lesion. **k** The mucosal incision is widened to the right and left. **l** A whole circumferential incision is made. **m** Submucosal dissection is performed. **n** En bloc resection is achieved.

A mucosal incision was made on the distal side to mark the incision limit. This was followed by a proximal mucosal incision and a complete circumferential incision. Finally, submucosal dissection was continued, and a complete en bloc resection was performed ( Fig. 3 **i–n** ). Additional gel was injected as needed to obtain a clear view of the submucosal layer.

In conclusion, low-pressure endoscopy with water and gel immersion may help endoscopists overcome poor endoscope operability in procedures involving a UDS. This approach may further reduce the patient’s suffering, stabilize their condition, and enable safe resection of UDS lesions.

Endoscopy_UCTN_Code_TTT_1AO_2AG_3AD

## Notes

Endoscopy E-Videos https://eref.thieme.de/e-videos
*E-Videos* is an open access online section of the journal *Endoscopy* , reporting on interesting cases and new techniques in gastroenterological endoscopy. All papers include a high-quality video and are published with a Creative Commons CC-BY license. Endoscopy E-Videos qualify for HINARI discounts and waivers and eligibility is automatically checked during the submission process. We grant 100% waivers to articles whose corresponding authors are based in Group A countries and 50% waivers to those who are based in Group B countries as classified by Research4Life (see: https://www.research4life.org/access/eligibility/ ). This section has its own submission website at https://mc.manuscriptcentral.com/e-videos .
